# Supplementary material for: Mechanical stress contributes to the expression of the STM homeobox gene in Arabidopsis shoot meristems
Source: eLife. 2015 Dec 1;4:e07811. doi: 10.7554/eLife.07811 (PMC4666715; doi:10.7554/eLife.07811)
Supplement: Supplementary file 1. — DOI: http://dx.doi.org/10.7554/eLife.07811.031 [file elife-07811-supp1.docx]

**Table 1.** **Summary of the number of replicates for each mechanical test**

|  | Number of repetitions | | | |
| --- | --- | --- | --- | --- |
|  | Ablations | Compressions | Controls | isoxaben-treated |
| *pSTM::CFP-N7* | >30 | 8 | >30 | 7 |
| *pBOUND::GFP* | 12 | 11 | 10 | 20 |
| *DII-Venus-N7* | 21 | 10 | 15 | 10 |
| *pPID::CFP-N7* | 13 |  | 6 |  |
| *pPID>>GFP* | 7 |  | 14 | 11 |
| *pin1-6 pBOUND::GFP* | 14 |  | 9 |  |
| *pin1-6 DII-Venus-N7* | 11 |  | 8 |  |
| *pSTM::CFP-N7* | 10 |  | 6 |  |
| *pCUC1::CUC1-GFP* | 11 |  | 8 |  |
| *pCUC3::CFP* | 20 |  | 16 |  |
| *ISH STM mRNA* | 9 |  | 7 |  |
